# The impact of incorporating surgical simulation into trichiasis surgery training on operative aspects of initial live-training surgeries

**DOI:** 10.1371/journal.pntd.0011125

**Published:** 2023-04-04

**Authors:** Emily W. Gower, Amir B. Kello, KH Martin Kollmann, Shannath L. Merbs, Alemayehu Sisay, Demissie Tadesse, Wondu Alemayehu, Nigel Pedlingham, Richard S. Dykstra, James E. Johnson

**Affiliations:** 1 Departments of Epidemiology and Ophthalmology, University of North Carolina at Chapel Hill, Chapel Hill, North Carolina, United States of America; 2 World Health Organization, Regional Office for Africa, Brazzaville, Republic of Congo; 3 CBM International, Nairobi, Kenya; 4 Department of Ophthalmology and Visual Sciences, University of Maryland School of Medicine, Baltimore, Maryland, United States of America; 5 Orbis Ethiopia, Addis Ababa, Ethiopia; 6 The Fred Hollows Foundation, United Kingdom; 7 Wake Forest School of Medicine, Winston-Salem, North Carolina, United States of America; Yale University School of Medicine, UNITED STATES

## Abstract

**Background:**

While surgical simulation is regularly used in surgical training in high-income country settings, it is uncommon in low- and middle-income countries, particularly for surgical training that primarily occurs in rural areas. We designed and evaluated a novel surgical simulator for improving trachomatous trichiasis (TT) surgery training, given that trichiasis is mostly found among the poorest individuals in rural areas.

**Methodology/Principal findings:**

TT surgery programs were invited to incorporate surgical simulation with a new, high fidelity, low-cost simulator into their training. Trainees completed standard TT-surgery training following World Health Organization guidelines. A subset of trainees received three hours of supplemental training with the simulator between classroom and live-surgery training. We recorded the time required to complete each surgery and the number of times the trainer intervened to correct surgical steps. Participants completed questionnaires regarding their perceptions. We also assessed trainer and trainee perceptions of surgical simulation training as part of trichiasis surgery training.

22 surgeons completed standard training and 26 completed standard training plus simulation. We observed 1,394 live-training surgeries. Average time to first live-training surgery completion was nearly 20% shorter the simulation versus the standard group (28.3 vs 34.4 minutes; p = 0.02). Trainers intervened significantly fewer times during initial live-training surgeries in the simulation group (2.7 vs. 4.8; p = 0.005). All trainers indicated the simulator significantly improved training by allowing trainees to practice safely and to identify problem areas before performing live-training surgeries. Trainees reported that simulation practice improved their confidence and skills prior to performing live-training surgeries.

**Conclusions:**

A single high-fidelity surgical simulation session can significantly improve critical aspects of initial TT surgeries.

## Author summary

Approximately 1.7 million people globally have an eyelid condition called trachomatous trichiasis (TT) resulting from repeated ocular bacterial infections that caused the eyelid to scar and turn in so that the eyelashes rub against the eye. TT can be corrected through surgery. However, TT is mostly found in areas with limited health care resources. Thus, nurses are trained how to perform the surgery. Historically, training has been limited to one week of classroom training followed by three weeks of live-surgery practical training. This study describes a surgical simulator the authors developed to improve the training process. After a three-hour training with the HEAD START simulator, trainees required less assistance from the trainer during live-patient surgical practice and performed surgery faster than those not trained on HEAD START.

## Introduction

In 2022, approximately 1.7 million individuals globally were thought to be living with trachomatous trichiasis (TT), a condition characterized by entropion and in-turned eyelashes that abrade the eye [1]. TT requires early identification and surgical management to prevent visual impairment. The World Health Organization (WHO) has set a goal of eliminating trachoma as a public health problem, with provision of sight-saving surgery to all who need it as one of the cornerstones of elimination. Significant scale up is underway to address the surgical backlog.

Addressing this backlog has challenges. TT is a public health problem primarily in rural areas of low- and middle-income countries (LMICs), where access to highly-trained surgeons is limited. Previous research demonstrated that eye care workers can perform TT surgery with success rates similar to ophthalmologists [2], broadening the pool of individuals who can tackle the backlog. However, surgical-outcome quality varies, with post-operative TT (PTT) rates ranging from 10% to >60% within one year [3–9]. Poor outcomes have significant programmatic impact, as affected individuals need further management, and their negative experiences may deter others from seeking surgical services. In order to reach the WHO elimination target of a prevalence of TT “unknown to the health system” of <0.2% in adults aged ≥15 years, significant improvement in TT-surgery quality is needed, with substantial focus on improving TT-surgery training and monitoring [10].

In most settings, national trachoma control programs select TT-surgery trainees from a pool of individuals with a minimum of nursing-level training and experience performing episiotomy and/or wound suturing, such as general nurses in rural health clinics. When this project began, TT surgery trainees completed a one-week classroom training followed by 2–4 weeks of live-surgery training, depending on class size and national program guidelines. If successful, trainees were certified to perform TT surgery independently with continued, occasional supportive supervision. Typically, trainees proceeded directly from classroom training to live-surgery training. This is in stark contrast to surgical-training programs in high-income-country settings where surgical simulation devices and other modes of repetitive skills training and enhancement have become commonplace prior to live surgery [11–14]. Taking lessons from these programs, we developed and tested a surgical simulation device to aid in training new TT surgeons. We undertook this study to assess the impact of introducing the simulator into TT surgery-training programs and to assess potential improvements in the training quality, particularly focusing on intraoperative characteristics and trainee and trainer perceptions.

## Methods

### HEAD START surgical simulation device development

**Fig 1:**
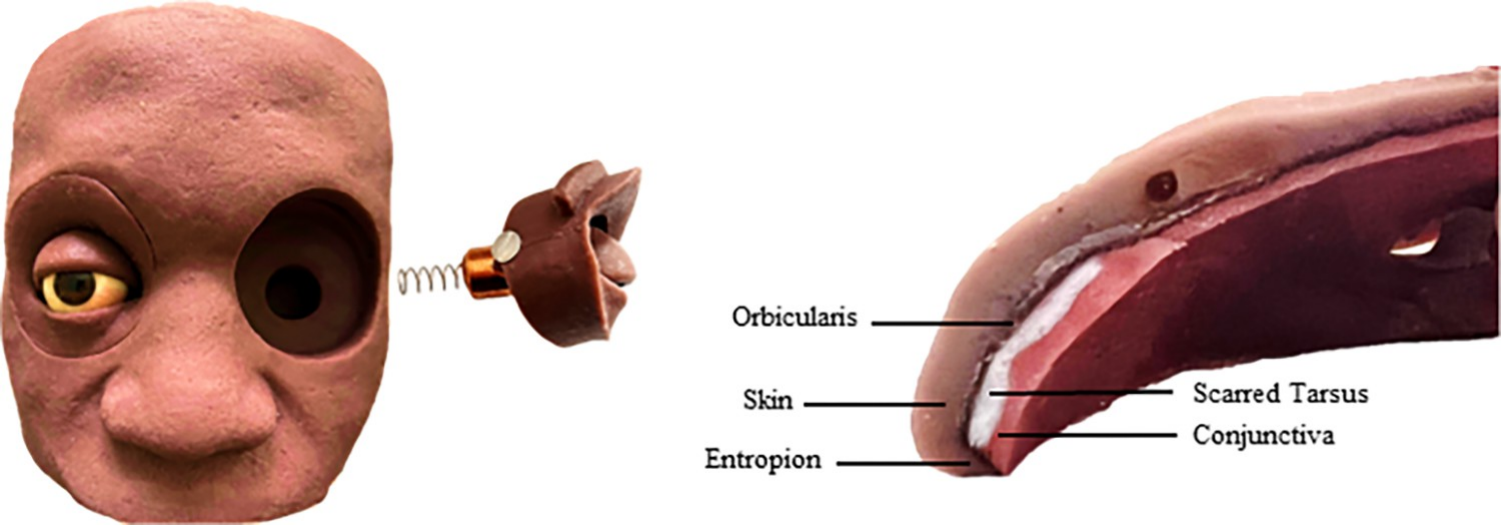
HEAD START Surgical Simulator base with removable orbits (left) and eyelid cartridge cross-section showing entropic eyelid margin and layers of eyelid (right).

**Fig 2:**
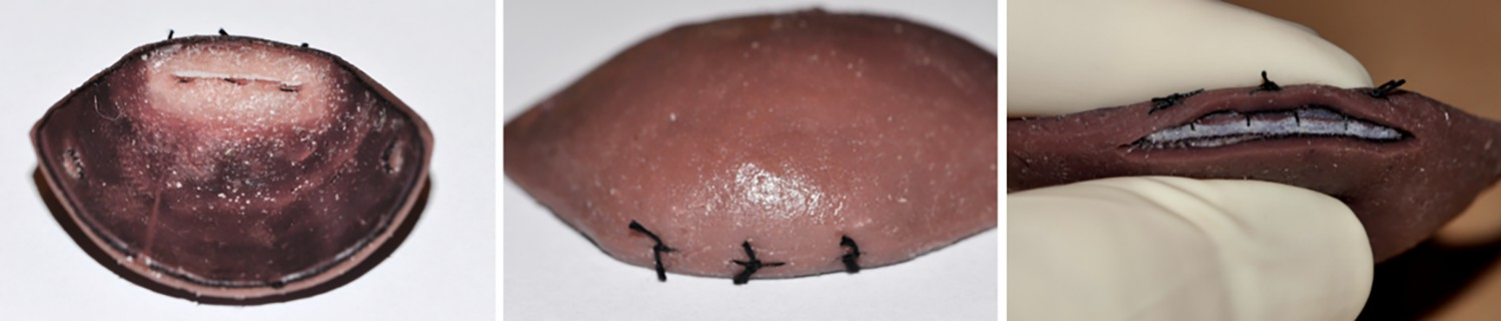
Eyelid cartridges can be removed to facilitate evaluation incision height, suture spacing, and suture placement in the tarsus.

The Human Eyelid Analogue Device for Surgical Training and Skills Reinforcement in Trichiasis (HEAD START) is a mannequin-based, high-fidelity simulation device consisting of a reusable silicone base, with removable sockets for inserting disposable eyelid cartridges on which TT surgery can be practiced (**Fig 1**). The eyelid cartridges are designed to mimic the main layers of the human upper eyelid: skin, muscle, tarsus, and conjunctiva. However, the eyelid margin of the cartridges demonstrates entropion to mimic the anatomy of an eyelid with TT. The handmade device currently costs $450 for the base and orbits, while eyelid cartridges are $12.50. The device can be used for both common TT surgery procedures, Bilamellar Tarsal Rotation (BLTR) and Posterior Lamellar Tarsal Rotation (PLTR), also called modified Trabut [15]. All key procedure steps can be performed on the device, except injecting anesthetic, since the synthetic eyelid layers don’t hold fluid. HEAD START provides the opportunity to perform surgery in an environment where trainees can learn and practice the procedure on an inanimate object without risk of patient harm. A key benefit is that the eyelid cartridge can be removed easily and inspected for incision and suture placement (**Fig 2**).

Prior to this study, we developed a standardized HEAD START training protocol by testing and refining training approaches and eliciting feedback from trainers and trainees. The HEAD START training protocol involves a three-hour, one-on-one session with a trainer and trainee. The full HEAD START training protocol is described in the globally-utilized Training Trichiasis Surgeons for Trachoma Elimination Programs Manual [16]. In brief, HEAD START training begins with the trainer demonstrating how to prepare the surgical field and perform each step of the surgery. The trainer and trainee then perform the procedure in a stepwise fashion, with the trainer performing the first step on one eyelid, followed by the trainee performing the same step on the contralateral eyelid. Next, the trainee performs the full procedure on a new cartridge. Finally, the trainer observes one complete surgery, noting areas of success and areas needing improvement. The trainer and trainee review the results and decide whether the trainee is ready to start live-surgery training or if she/he would benefit from additional simulation practice, either through another complete session or targeted practice to improve a specific step, such as incision or suture placement. We utilized this approach for the study described herein.

### Study population and framework

At the study start, the BLTR procedure was the standard in the study region. Thus, all trainees learned how to perform BLTR surgery using the TT clamp [17]. The standard training followed the WHO Yellow Manual training protocol [15], which involved one week of didactic training followed by 2–4 weeks of live-surgery training. All trainees completed the same standard training protocol. Individuals assigned to HEAD START training also received simulation training after the classroom training but before live-surgery training. The five HEAD START trainers were ophthalmologists or cataract surgeons who we previously trained to be HEAD START trainers. The live-surgery training was conducted by two ophthalmic nurses with extensive experience performing TT surgery.

We conducted this study over a two-year period in Ethiopia in collaboration with two non-governmental development organizations (NGDO). These NGDOs led four training sessions for new surgeons, and all trainees were invited to participate. Zonal health officials identified the nurses to be trained. The Fred Hollows Foundation (FHF) conducted two training sessions in Woliso and Ambo, Oromia. In the first session, four of the 13 trainees were randomly selected to receive HEAD START training. To create the assignments, we recorded all trainees’ names in an Excel sheet and used the random number generator function to assign a value between 0 and 1 to each name. We then selected the four lowest values to be the HEAD START trainees. The statistician creating the Excel program did not know any of the trainees. In the second session, all 13 trainees followed the standard training protocol. Orbis Ethiopia conducted two training sessions in Welkite and Hosannah, Southern Nations, Nationalities, and Peoples’ region (SNNPR). All 22 trainees in the Orbis-led sessions received HEAD START training.

### Ethical clearance and consent

The institutional review boards at the University of North Carolina and Wake Forest School of Medicine, and the ethical clearance committees for the Southern Nations, Nationalities, and Peoples’ region and Oromia Health Bureaus and the Zonal Health Office for Southwest Shewa approved this study. The project followed the tenets of the Declaration of Helsinki. All trainees gave written consent to participate.

### Data collection

#### Live-surgery training

A trained data collector observed the trainees perform live surgeries throughout the training program. They recorded the time the trainee-surgeon injected anesthetic, when the TT clamp was placed, and when the last suture was tied. In addition, they recorded the number of times the trainer intervened. As with any training program, the trainer provided regular verbal feedback throughout the surgeries. Thus, an intervention was defined as the trainer needing to step in and physically correct or adjust something that the trainee had done, such as extending the incision or replacing a suture.

#### Questionnaires regarding HEAD START’s role in training

During the Welkite and Hosannah HEAD START training sessions (n = 22 total), at the end of live-surgery training, each HEAD START trainee completed a questionnaire (see S1 Text) regarding their perceptions of HEAD START and what steps were challenging on the simulator and during live-surgery training. Additionally, each trainer completed a questionnaire that elicited information on what was easy or challenging for the trainee at the start of the training, whether the trainee was able to overcome the challenges, the impact of simulator practice, and whether HEAD START facilitated training and skills acquisition. Finally, two of the five HEAD START trainers completed an overall assessment of HEAD START and provided feedback on areas where the approach might benefit from modification.

### Analysis

We calculated time to complete each surgery as the time that the last suture was tied minus the time that the injection was started. We calculated the time required for injection and clamp placement as the time the clamp was locked minus the time the injection was started. We calculated the time required for incising and suturing by subtracting the time the clamp was placed from the time the last suture was tied. We used means, medians and ranges to describe each characteristic. We used t-tests to compare differences in means between groups, and we report Satterthwaite p-values. All analyses were conducted using SAS software version 9.4 [2002–2012] SAS Institute Inc.

## Results

A total of 48 trainees participated; 22 trainees completed the standard training approach and 26 underwent training incorporating HEAD START. Most trainees were male (81%) and the majority were aged <30 years. All trainees were nurses from rural health posts who had experience with basic suturing of wounds but did not have any prior surgical experience.

### Live-surgery training

We observed 1,394 training surgeries. Each surgeon performed ≥20 surgeries during their training (mean = 29; max = 41). The average time for a trainee to complete the first live-training surgery was nearly 20% shorter for HEAD START trainees versus standard trainees (28.3 minutes versus 34.4 minutes; p = 0.02, **Table 1**). As expected, the time for injection was the same for both groups (4.7 minutes). During their first surgery, HEAD START trainees required roughly half the number of trainer interventions compared to standard trainees (2.7 vs 4.8; p = 0.0046). By the 15^th^ surgery per trainee, the number of interventions was very low and the same for both groups.

**Table 1:** Characteristics of First Training Surgeries According to Training Group.

	Standard Training Mean (Std. Dev.)	HEAD START Mean (Std. Dev.)	P-value
N	22	26	
Total surgery duration, minutes	34.4 (6.8)	28.3 (9.9)	0.02
Time to inject and insert clamp, minutes	4.7 (4.0)	4.7 (3.9)	0.36
Time to incise and suture, minutes	30.3 (5.8)	23.1 (8.9)	0.002
Trainer interventions, number	4.8 (2.6)	2.7 (2.2)	0.005

### Trainer and trainee feedback

Trainer questionnaires were available for 19 individual HEAD START training sessions. The most common task that trainers indicated trainees completed easily at the start of training was the ability to place the TT clamp (79%), followed by the ability to make a straight incision (42%; **Table 2**). Trainers indicated that initially, all trainees struggled to evenly space their sutures, and just over half (58%) were not able to make proper passes with the needle. HEAD START trainees required 3–6 total practice surgeries on the simulator before the trainer felt they were ready to start live-training surgery. All trainers indicated that simulation training was beneficial in preparing trainees to perform live surgery. The trainers indicated that repeated practice allowed trainees to become adequately skilled in each of the necessary steps. When asked to provide their final impression regarding how HEAD START facilitated the training process, trainers indicated it was useful for all trainees. Trainers noted that a key benefit of the simulator is that it allows trainees to learn from their mistakes and to develop their confidence before performing live surgery. Of note, one trainer also emphasized that this tool was particularly useful for individuals who were overly confident in their skills by allowing identification of actual weaknesses before operating on live patients. Finally, trainers noted that HEAD START allowed for focused practice on the steps that were most challenging for a given trainee.

**Table 2:** Trainer Assessment of Trainees on HEAD START at Start of Training Session.

	Trainer Assessment of Trainee on HEAD START*	
Task	Easy N(%)	Difficult N(%)
Placing the TT clamp	15 (79)	4 (21)
Handling instruments	7 (37)	5 (26)
Straight incision	8 (42)	9 (47)
Taking proper bites with the needle	3 (16)	11 (58)
Evenly spacing sutures	0 (0)	19 (100)
Tying knots	3(16)	3 (16)

*Easy and challenging columns do not add to 100% because trainers were asked open-ended questions regarding what was easy and what was challenging. Thus, trainers did not always report each task.

Similarly, trainees consistently provided positive feedback about the experience of practicing with HEAD START prior to undergoing live-surgery training (**Tables 3 and 4**). All 21 trainees who completed questionnaires recommended HEAD START should be a standard part of TT-surgery training. Table 4 summarizes the open-ended feedback provided by the trainers and trainees. Among these, seven trainees specifically stated that it improved their confidence, while others indicated that it helped improve their understanding of the procedure steps and eyelid anatomy and prepared them to perform live surgery successfully by learning from their mistakes. When asked what modifications should be made to the device, four requested making the tarsal plate less rigid, while the remainder indicated no changes were needed.

**Table 3:** Trainee Self-Assessment of Difficulty in Performing Tasks at the Beginning of HEAD START training and Live-training Surgeries.

Task	Difficult at the beginning of HEAD START Training N(%)	Difficult on First Live Surgery N(%)
Placing the TT clamp	8 (38)	6 (29)
Handling instruments	7(33)	4 (19)
Straight incision	10 (48)	7(33)
Taking proper bites with the needle	11 (52)	7(33)
Tying knots	2 (10)	1(5)

**Table 4:** Open-ended Trainee Feedback.

Question/responses	Number of Trainees (out of 21)
**Do you recommend HEAD START for future training programs?**	Yes = 21, No = 0
**Reasons for recommending HEAD START:**	
Improves confidence	7
Prepares trainees for live surgery	6
Increases instrument handling skills	4
Improves understanding of eyelid anatomy	2
Very important, appreciate you, best one, helps me to become special surgeon	11
**What modifications would you make to the device or training program?**	
Make the tarsus less stiff	4
Change the size of the eyelid part	2
Increase amount of time spent on HEAD START	1
No changes, it is good as is	15

During the summary questionnaires completed by two trainers (**Table 5**), one stated “It was very helpful to build trainees’ confidence, as previously they had to start learning how to do surgery on live patients with much anxiety. With the HEAD START, they do not need to worry about doing harm to a patient. They also learn the steps involved in the surgery, which helps save time during the first live-training surgery, as the trainer does not need to show them the steps. The trainees demonstrate more confidence during live surgery.” The other trainer provided similar comments and said “Above all they came with confidence and eagerness to quickly learn doing surgery on a live patient.”

**Table 5:** Open-ended Trainer Feedback on the Overall HEAD START Process.

Question/responses	Number of responses (out of 2)
**Was HEAD START helpful in preparing new surgeons for their first live-training surgery (yes or no)?**	Yes = 2
**Open-ended responses on why or why not:**	
Familiarizes trainees with the procedure	2
Develops confidence, reduces anxiety	2
Trainees did not have to worry about harming patients	1
Trainees know what is expected of them during surgery	1
Makes life easier on first live-training surgeries	2
**What changes would you make to the simulator?**	
Reduce tarsus stiffness	2

## Discussion

This study demonstrated significant improvements in standard quality metrics for initial live TT surgeries performed by trainees who completed a three-hour surgical simulation training with HEAD START prior to conducting live surgery during training. Initial live surgeries performed by HEAD START trainees required fewer trainer interventions, and HEAD START trainees’ first surgeries were faster than the standard group. These findings indicate significant benefit from using surgical simulation in TT-surgery training.

HEAD START trainees performed their first live surgery approximately 20% faster than standard trainees (34.4 vs. 28.3 minutes; p = 0.02). This six-minute change may be clinically significant because it is important to limit the duration of hemostasis, which is achieved by utilizing the TT clamp or hemostats, in order to minimize risk of long-term damage to the eyelid. Additionally, reducing the surgical time ultimately benefits the patient by reducing the risk that the anesthesia and epinephrine will wear off during the procedure. This change in time is similar to a simulation study that reported a 29% reduction in time required for laparoscopic gallbladder dissection among residents who received simulation training [18]. Numerous other studies have also demonstrated reduced suturing time following practice on a simulator [19–21].

More importantly, trainers intervened on surgeries performed by standard surgeons twice as often as they intervened on surgeries by HEAD START trainees. Ensuring high-quality surgery is important for multiple reasons, and this measurement indicates improved surgical quality because it shows the trainer was more comfortable with the HEAD START trainees’ skill level. As a result, patients operated on by trainees with prior simulation practice may be less likely to experience complications that are more common when a trainee performs surgery, such as division of the eyelid margin or problems resulting from improper incision placement. Previous work also has reported reduced need for trainers to intervene when a trainee has received simulation training prior to live surgery [18].

Simulation devices that bridge the gap between classroom and live surgery have become prevalent in surgical training in high-income countries [12,18,22,23]. Recent studies of cataract surgery training have shown benefits of simulation devices utilized before surgery is performed on live patients. A study of a capsulorhexis simulation concluded that there is substantial benefit from simulation training in reducing the risk of problems during cataract surgery [24]. Specifically, a simulation model for capsulorhexis reduced the rate of errant continuous, curvilinear capsulorhexes three-fold. A review of simulators for cataract surgery also concluded that performance on surgical tasks was significantly improved by simulation training [25]. Furthermore, a recent multi-country qualitative study of trainee ophthalmologists and surgeon educators reported that simulation is perceived as an important and valuable educational model for surgery training in sub-Saharan Africa [26]. The HEAD START simulator fills an important void in trichiasis surgery training in Africa.

Previous research highlights three important components of good simulators: validity, reliability, and feasibility [12]. The simulator evaluated here meets all of these criteria. It is used to practice the critical steps of performing high-quality TT surgery: placing the clamp or Trabut plate, making the incision and placing sutures. Afterwards, the instructor can remove the eyelid cartridge from the simulator base, allowing additional assessment of the surgery quality. We designed the cartridges to mimic a human eyelid with TT, so that cutting through the cartridge feels similar to cutting through the scarred tarsus. Additionally, the eyelid cartridges mimic the thickness and layers of the human eyelid, using materials that hold sutures without tearing. The simulator is highly portable, allowing surgeons to move directly between simulation and live surgery on the same day, and to return to the simulator at any time to practice as needed. This makes simulation training feasible virtually anywhere, without the need for electricity or sophisticated machinery.

Prior studies also emphasized the need for using surrogate measures to assess quality since surgical outcomes can take a long time to develop, and previous simulation studies have used similar measures to ours [12,23]. One study assessed surgery residents on anastomotic simulators. Like the HEAD START simulator, trainees were assessed for appropriate placement and spacing of sutures as well as knot tying and instrument handling, and they reported improved scores on each of these after simulation training [23].

This study goes beyond evaluation on the simulator and shows improvement in a real-world setting. In addition to demonstrated success using measurable, quantitative outcomes, this study showed strong support for integration of surgical simulation into TT surgery training. All trainees felt that simulation training improved their training experience, with many citing increased confidence and understanding of the surgical steps following simulation training. Additionally, all trainers indicated that simulator training was beneficial, both for training surgeons and for identifying those who need closer supervision during live surgery training. This could have significant implications for surgical programs, as it is likely to reduce the risk of clinically important surgical mistakes, such as cutting through the eyelid margin or puncturing the globe. Additionally, integrating simulation training before live-surgery training allows for more scrutiny before deciding on a trainee’s suitability for live-surgery training, which should reduce risk to patients. While our study shows statistically significant results, it is important to note that our findings are based on 48 trainees. Future studies should involve larger sample sizes and would benefit from masking those who collected the study outcomes.

We have developed a standardized tool for assessing TT surgeons using surgical simulation that is available globally as part of the best practice guidelines for TT surgery training [16]. Additionally, WHO now recommends use of a surgical simulator for all new and refresher TT-surgery training [27]; currently, HEAD START is the only surgical simulator available for this purpose. This simulator also is likely to be beneficial in other ophthalmic-training settings, both in LMIC countries and high-income countries. It recently was modified to train US-based ophthalmology residents on margin-involving eyelid laceration repair. Ophthalmic-residency programs may want to consider additional areas for resident training using this simulator.

In summary, this study demonstrated significant improvement in quantitative measures of surgical quality. Trainees and trainers expressed strong interest in its use for TT-surgery training. Future work should evaluate the impact of HEAD START training on longer-term surgical outcomes.

## Supporting information

S1 Text Trainer and Trainee Questionnaires. (PDF)
