# Supplementary material for: The impact of incorporating surgical simulation into trichiasis surgery training on operative aspects of initial live-training surgeries
Source: PLoS Negl Trop Dis. 2023 Apr 4;17(4):e0011125. doi: 10.1371/journal.pntd.0011125 (PMC10112793; doi:10.1371/journal.pntd.0011125)
Supplement: S1 Text — (PDF) [file pntd.0011125.s001.pdf]

Name: ______________________________

Trainee Questionnaire regarding HEAD START

1) Did HEAD START help you prepare for live surgery? ☐Yes ☐No

2) Did it help you with:

a. Learning how to handle instruments? ☐Yes ☐No
b. Knowing the steps of the surgery? ☐Yes ☐No
c. Becoming more confident as a surgeon? ☐Yes ☐No
d. Understanding the eyelid anatomy? ☐Yes ☐No

3) When using HEAD START at first, was

handling instruments ☐easy or ☐difficult
placing the clamp ☐easy or ☐difficult
making an incision ☐ easy or ☐difficult
placing sutures ☐ easy or ☐difficult
tying sutures ☐easy or ☐difficult

3) For your first live surgery, was

maintaining sterility ☐easy or ☐difficult
cleaning the eyelid ☐easy or ☐difficult
injecting ligdocaine ☐easy or ☐difficult
handling instruments ☐easy or ☐difficult
placing the clamp ☐easy or ☐difficult
making an incision ☐easy or ☐difficult
placing sutures ☐easy or ☐difficult
tying sutures ☐easy or ☐difficult

7) Do you recommend that HEAD START be used for future training sessions with new surgeon trainees? Why or why not?

8) What changes to the HEAD START training process would you recommend?

9) What changes to the mannequin do you recommend?

10) Was making an incision easier on the mannequin or in live surgery?

☐Mannequin
☐Live surgery

11) Was suturing easier on the mannequin or live surgery?

☐Mannequin
☐Live surgery

Trainer Summary of Trainee Skills Assessment

1) Which steps were easy/simple for the trainee at the beginning of HEAD START?
1. Placing the TT clamp properly, holding and manipulating instruments, Making an incision parallel to lid margin.

2) Which steps were challenging for the trainee at the beginning of HEAD START?

3) For the steps that were challenging, how did the trainee progress during HEAD START training, and to what level of satisfaction for recommending him for live surgery? (What was your impression of final outcome on the issues that were a challenge at the beginning)?

4) How much has repeated practice with HEAD START improved the areas that he was challenged with at the beginning? Do you think that HEAD START positively influenced the skills improvement during the training process?

5) What is your final impression of how HEAD START facilitated the training process or skills acquisition of the trainee?

Legends

Figure 1: HEAD START Surgical Simulator base with removable orbits (left) and eyelid cartridge cross-section showing entropic eyelid margin and layers of eyelid

Figure 2: Eyelid cartridges can be removed to facilitate evaluation incision height, suture spacing, and suture placement in the tarsus.
